# Effective Mode Approximation for Probabilistic Verification of Collective Hamiltonians in Large Continuous-Variable Quantum Systems

**DOI:** 10.3390/e28050514

**Published:** 2026-05-02

**Authors:** José R. Rosas-Bustos, Jesse Van Griensven Thé, Roydon Andrew Fraser, Nadeem Said, Sebastian Ratto Valderrama, Mark Pecen, Alexander Truskovsky, Andy Thanos

**Affiliations:** 1 Department of MME, University of Waterloo, Waterloo, ON N2L 3G1, Canada; jesse.the@uwaterloo.ca (J.V.G.T.); rafraser@uwaterloo.ca (R.A.F.); nadeem.said@uwaterloo.ca (N.S.); 2 LAKES Environmental Research Inc., Waterloo, ON N2L 3L3, Canada; 3 Applied Quantum Technologies (AQT) Initiative, Columbia, MD 21046, USA; srattova@uwaterloo.ca (S.R.V.); mark@eigenq.com (M.P.); 4 EigenQ, Inc., Austin, TX 78701, USA; alex@eigenq.com; 5 Department of ECE, University of Waterloo, Waterloo, ON N2L 3G1, Canada; 6 Cisco Systems, Inc., San Jose, CA 95134, USA; athanos@cisco.com

**Keywords:** effective mode approximation, continuous-variable quantum systems, multi-qu-mode systems, collective quadratures, collective Hamiltonian verification, time-resolved homodyne detection, Gaussian simulations, model selection, residual analysis, scalable quantum characterization

## Abstract

The Effective Mode Approximation (EMA) is a verification-oriented framework for characterizing collective Hamiltonian dynamics in large continuous-variable (CV) quantum systems from experimentally accessible collective measurements. Rather than reconstructing a full mode-resolved Hamiltonian, EMA maps the observed dynamics onto a canonically normalized collective mode and tests whether summed quadrature trajectories are consistent with an effective harmonic description. We validate EMA using time-resolved homodyne sampling in Gaussian simulations of ring-coupled multi-qu-mode optical systems with N=8,16,32, and 64 modes. One-tone and two-tone sinusoidal models, selected using the Akaike Information Criterion (AIC), recover a stable dominant collective frequency across system size and produce residuals that remain centred near zero. The results show that EMA can verify dominant collective behaviour with a fixed number of effective parameters even when full microscopic reconstruction is impractical. EMA is therefore best understood not as a full-state ansatz, but as a low-overhead tool for validating collective dynamics under realistic measurement constraints in scalable CV hardware.

## 1. Introduction

Continuous-variable (CV) quantum systems provide a powerful and experimentally mature platform for quantum information processing, leveraging Gaussian quantum states and quadrature-based measurements to encode and manipulate information [1]. Gaussian states, whose Wigner functions are completely specified by first and second quadrature moments, are especially important in this setting. By operating on continuous degrees of freedom of the electromagnetic field, CV architectures support key quantum protocols, including quantum communication, sensing, and cryptographic primitives, while offering practical advantages in scalability, measurement bandwidth, and hardware integration when compared to discrete-variable approaches [2].

Modern CV platforms increasingly rely on multi-qu-mode architectures, where many optical or bosonic modes interact within a shared physical system, such as an optical cavity or integrated photonic circuit [3]. In these settings, understanding and validating the effective Hamiltonian governing collective dynamics is essential for system characterization, stability assessment, and verification of correct device operation. Time-resolved homodyne detection provides a natural and minimally invasive mechanism for monitoring such systems, yielding access to collective quadrature observables that encode global system behaviour [4].

However, conventional Hamiltonian reconstruction techniques typically require explicit, mode-resolved modelling of individual qu-modes and their pairwise interactions. As system size and entanglement increase, this approach rapidly becomes impractical due to exponential scaling of model parameters and computational cost. Moreover, in highly entangled or symmetric systems, detailed access to individual mode dynamics is often neither experimentally accessible nor necessary: collective observables dominate the measurable behaviour and operational relevance of the device.

To address these challenges, we introduce the **Effective Mode Approximation (EMA)**, a scalable framework for collective Hamiltonian characterization in large multi-qu-mode systems. Rather than reconstructing the full microscopic Hamiltonian, EMA models the system as a single effective collective mode whose dynamics are inferred directly from summed quadrature measurements. This approach enables efficient extraction and verification of dominant collective parameters, such as effective oscillation frequencies, without requiring mode-by-mode access or detailed microscopic knowledge of the underlying couplings.

EMA is particularly well-suited to engineered CV platforms where symmetry, collective coupling, or strong entanglement leads to coherent global behaviour. In this work, we validate the EMA framework using time-resolved Gaussian simulations of ring-coupled qu-mode systems with N=8,16,32, and 64 modes. By fitting single- and multi-tone harmonic models to collective quadrature trajectories and analyzing residuals across repeated runs, we demonstrate that EMA provides a robust probabilistic validation of collective dynamics, with residuals consistent with statistical noise and stable parameter estimates across increasing system size.

Importantly, this approach emphasizes verification and validation rather than exact reconstruction, aligning with the needs of practical quantum engineering and device characterization. EMA offers a computationally lightweight method for confirming correct collective behaviour in high-dimensional CV systems, making it directly relevant to scalable quantum technologies, continuous-variable quantum computing, and emerging verification pipelines for complex quantum hardware [5,6].

We emphasize that, for Gaussian states evolving under linear dynamics, EMA is not intended as a substitute for standard covariance-matrix methods when complete mode-resolved information is available. Its role is narrower: to provide a lightweight inference and validation procedure when only collective homodyne-accessible observables are available. The objective is to test consistency with an effective collective model rather than reconstruct the full microscopic dynamics.

More specifically, the contribution of this work is fourfold. First, we formulate EMA in terms of a canonically normalized collective mode, which makes the effective single-oscillator Hamiltonian physically well-posed. Second, we develop a validation pipeline based on homodyne-accessible summed quadratures rather than mode-resolved reconstruction. Third, we combine one-tone/two-tone fitting, AIC-based model selection, and residual analysis to assess when a single collective mode suffices. Fourth, we clarify the scope of EMA as a reduced-order method for validating collective dynamics, not as a full wavefunction-reconstruction ansatz.

This paper presents the EMA framework, details its implementation using time-resolved homodyne-accessible observables, and demonstrates its effectiveness through numerical validation across increasing system sizes. The results highlight EMA as a practical tool for scalable characterization of collective quantum dynamics in multi-qu-mode continuous-variable systems.

## 2. Literature Review

Approximation and reduction techniques have long played a central role in quantum mechanics, particularly for systems involving many degrees of freedom or strong correlations. In quantum optics and continuous-variable (CV) quantum information, such methods are essential for rendering complex multi-mode dynamics computationally tractable while preserving the dominant physical behaviour of the system.

A broad class of work has focused on effective Hamiltonian descriptions, where microscopic details are partially coarse-grained in order to capture dominant collective dynamics. In cavity quantum electrodynamics, optomechanics, and photonic lattice systems, effective Hamiltonians are routinely employed to describe collective coupling effects, renormalized mode frequencies, or emergent normal modes without explicitly modelling all underlying interactions [4,7]. Similar approaches appear in studies of Bose–Einstein condensates, symmetric many-body systems, and Gaussian photonic networks, where collective observables provide a compact description of system-level behaviour [8,9]. These methods, however, typically retain an explicit multi-mode structure and are primarily used for analytical modelling rather than scalable validation or verification.

Related simplifications arise in the study of open quantum systems, where quasi-mode, effective bath, or Markovian approximations reduce environmental complexity by isolating dominant dynamical contributions [10,11]. While powerful for analyzing dissipation and decoherence, these techniques focus on dynamical reduction rather than direct inference from experimentally accessible collective measurements. As such, they are not designed to support efficient validation of large-scale multi-qu-mode devices based on aggregate observables.

In the context of continuous-variable quantum information, scalability concerns have motivated a growing interest in collective descriptions that emphasize global properties over individual mode dynamics. Measurement-based CV quantum computing, cluster-state architectures, and large Gaussian networks increasingly rely on collective quadrature measurements and global correlations to characterize system performance [3,5]. In these platforms, full mode-resolved Hamiltonian reconstruction is often impractical or experimentally inaccessible, motivating alternative approaches that focus on verifying collective behaviour rather than reconstructing microscopic interaction graphs [12,13].

A distinct but relevant line of research is provided by tensor-network methods, which seek efficient representations of many-body quantum states by exploiting entanglement structure. In particular, projected entangled pair states (PEPS) extend matrix–product–state ideas beyond one dimension, and continuous projected entangled pair states (cPEPS) extend the same logic to quantum fields [14,15]. These approaches are highly expressive when the goal is state representation or simulation, but they address a different task from EMA. EMA does not attempt to reconstruct or compress the full multi-mode wavefunction; instead, it asks whether experimentally accessible collective observables are consistent with a low-dimensional effective Hamiltonian. This distinction is important for assessing efficiency: tensor-network methods trade computational cost for representational richness, whereas EMA sacrifices microscopic completeness in exchange for direct, measurement-driven validation.

The Effective Mode Approximation (EMA) introduced in this work builds on these ideas while addressing a distinct gap in the literature. Rather than proposing a new microscopic model or renormalized Hamiltonian, EMA provides a verification-oriented framework that infers an effective collective dynamical description directly from time-resolved collective quadrature measurements. By modelling the observed dynamics as those of a single effective harmonic mode and validating this model through statistical fitting and residual analysis, EMA enables scalable characterization of large multi-qu-mode systems without requiring detailed access to individual modes or couplings.

In this respect, EMA complements the existing effective Hamiltonian and mode-reduction techniques by shifting the emphasis from theoretical reduction to practical validation. This distinction is particularly relevant for engineered CV quantum systems, where experimental constraints, symmetry, and strong correlations naturally privilege collective observables. As such, EMA contributes a practical and computationally efficient methodology for assessing collective Hamiltonian behaviour in large-scale continuous-variable quantum platforms.

## 3. Theoretical Framework for Effective Collective Hamiltonian Inference

This section outlines the theoretical framework underlying the Effective Mode Approximation (EMA) for characterizing the collective dynamics of multi-qu-mode continuous-variable (CV) systems using time-resolved homodyne detection. Rather than aiming for a complete microscopic reconstruction of all individual mode interactions, the framework focuses on inferring an effective collective dynamical description directly from experimentally accessible quadrature measurements. This perspective is particularly well-suited to large-scale, symmetric, or highly correlated CV systems, where collective observables dominate the measurable dynamics [1,2].

### 3.1. Hilbert Space Representation of Multi-Qu-Mode Systems

In continuous-variable quantum mechanics, each qu-mode *i* is associated with an infinite-dimensional Hilbert space $H_{i}$, spanned by the Fock states $\left\{|n_{i}\rangle \right\}$. The canonical quadrature operators ${\hat{x}}_{i}$ and ${\hat{p}}_{i}$ describe the field amplitude and phase of each mode and satisfy the canonical commutation relation

(1) $$[{\hat{x}}_{i},{\hat{p}}_{i}]=i\hbar .$$

This relation implies the Heisenberg uncertainty principle

(2) $$\Delta x_{i}\Delta p_{i}\geq \frac{\hbar }{2},$$

which sets a fundamental limit on the simultaneous knowledge of conjugate quadratures.

For a system comprising *N* qu-modes, the total Hilbert space is given by the tensor product

(3) $$H_{\mathrm{total}}=H_{1}⊗H_{2}⊗\cdots ⊗H_{N}.$$

A general joint quantum state may be expanded in the product Fock basis as

(4) $$|\Psi_{\mathrm{total}}\rangle =\sum_{n_{1},\ldots ,n_{N}}c_{n_{1},\ldots ,n_{N}}|n_{1}\rangle ⊗\cdots ⊗|n_{N}\rangle ,$$

where the basis vectors are tensor-product basis states, but the state itself is not generally separable. Correlations and entanglement across modes are encoded in the coefficient tensor $c_{n_{1},\ldots ,n_{N}}$. Equation (4) reduces to a direct product state only when these coefficients factorize as $c_{n_{1},\ldots ,n_{N}}=\prod_{i=1}^{N}c_{n_{i}}^{\left(i\right)}$. While this full description rapidly becomes intractable as *N* grows, many experimentally relevant observables depend primarily on collective properties rather than individual mode occupations.

### 3.2. Multi-Qu-Mode Hamiltonians and Collective Structure

The total Hamiltonian of a multi-qu-mode system can be written in the generic form

(5) $${\hat{H}}_{\mathrm{total}}=\sum_{i=1}^{N}\hbar \omega_{i}{\hat{a}}_{i}^{†}{\hat{a}}_{i}+\sum_{i\neq j}g_{ij}\left({\hat{a}}_{i}^{†}{\hat{a}}_{j}+{\hat{a}}_{i}{\hat{a}}_{j}^{†}\right),$$

where $\omega_{i}$ denotes the bare frequency of mode *i* and $g_{ij}$ characterizes inter-mode coupling. In practice, the precise values of $\omega_{i}$ and $g_{ij}$ may be unknown, time-dependent, or inaccessible, particularly in engineered photonic systems with many modes.

#### Symmetric and Strongly Correlated Regimes

In highly symmetric configurations, the coupling structure simplifies, with approximately uniform couplings $g_{ij}\approx g$. In this regime, it is natural to define collective ladder operators,

(6) $$\hat{A}=\sum_{i=1}^{N}{\hat{a}}_{i},$$

which allow the interaction Hamiltonian to be expressed in terms of collective excitations. Such collective descriptions arise naturally in models ranging from symmetric cavity arrays to collective spin–boson systems and optically coupled resonators [16].

Similarly, in the symmetric or near-symmetric Gaussian networks considered in this work, collective quadrature operators

(7) $${\hat{X}}_{\mathrm{total}}=\sum_{i=1}^{N}{\hat{x}}_{i},\;{\hat{P}}_{\mathrm{total}}=\sum_{i=1}^{N}{\hat{p}}_{i}$$

are the observables directly accessed by the homodyne protocol and therefore provide the most natural reduced coordinates for model validation [1,3]. When collective coupling dominates the observed response, the system’s evolution can be meaningfully characterized through these collective degrees of freedom even when individual mode dynamics remain unresolved.

### 3.3. Computational Motivation for Effective Descriptions

In the Gaussian and linear regime considered here, when the multi-mode Hamiltonian in Equation (5) is fully specified, forward evolution can indeed be carried out efficiently using covariance-matrix propagation or equivalent normal-mode methods [1,9]. The motivation for EMA is therefore not to replace these standard Gaussian tools. Rather, EMA addresses a different task: verification-oriented inference under restricted collective readout, where the experimentally available evidence consists of time-resolved measurements of summed quadratures rather than full mode-resolved access to all observables or microscopic parameters.

Under this access model, the practical challenge is not merely forward simulation, but also the measurement-access constraints, statistical overhead, and inference burden required to determine whether the observed dynamics are consistent with a low-dimensional collective description. EMA therefore seeks a system-level consistency test based on collective trajectories, model selection, and residual diagnostics, rather than unique recovery of the underlying microscopic Hamiltonian.

### 3.4. Effective Collective Hamiltonian Model

The collective observables introduced in Equation (7) are directly accessible via time-resolved homodyne detection. However, because they are defined as sums over *N* canonical pairs, they are not canonically normalized:

(8) $$[{\hat{X}}_{\mathrm{total}},{\hat{P}}_{\mathrm{total}}]=iN\hbar .$$

To define a well-posed single effective collective oscillator, we introduce the normalized collective quadratures

(9) $${\hat{X}}_{c}=\frac{1}{\sqrt{N}}\sum_{i=1}^{N}{\hat{x}}_{i},\;{\hat{P}}_{c}=\frac{1}{\sqrt{N}}\sum_{i=1}^{N}{\hat{p}}_{i},$$

which satisfy the canonical commutation relation

(10) $$[{\hat{X}}_{c},{\hat{P}}_{c}]=i\hbar .$$

In terms of experimentally measured summed observables, the relationship is a known scale factor:

(11) $$X_{\mathrm{total}}\left(t\right)=\sqrt{N}\;X_{c}\left(t\right),\;P_{\mathrm{total}}\left(t\right)=\sqrt{N}\;P_{c}\left(t\right).$$

Within EMA, the collective dynamics are modelled using an effective Hamiltonian of the form

(12) $${\hat{H}}_{\mathrm{eff}}=\frac{\hbar \Omega_{\mathrm{eff}}}{2}\left({\hat{X}}_{c}^{2}+{\hat{P}}_{c}^{2}\right),$$

where $\Omega_{\mathrm{eff}}$ is an effective collective frequency inferred from data. This Hamiltonian corresponds to a single harmonic oscillator governing the collective mode defined by Equations (9) and (10).

Under Equation (12), the normalized collective quadratures evolve as

(13) $$\begin{array}{cc} {\hat{X}}_{c}\left(t\right) & ={\hat{X}}_{c}\left(0\right)\cos\;\left(\Omega_{\mathrm{eff}}t\right)+{\hat{P}}_{c}\left(0\right)\sin\left(\Omega_{\mathrm{eff}}t\right), \end{array}$$

(14) $$\begin{array}{cc} {\hat{P}}_{c}\left(t\right) & ={\hat{P}}_{c}\left(0\right)\cos\;\left(\Omega_{\mathrm{eff}}t\right)-{\hat{X}}_{c}\left(0\right)\sin\left(\Omega_{\mathrm{eff}}t\right). \end{array}$$

These expressions provide a directly testable model against time-resolved homodyne measurements. In practice, the fitting and residual analysis may be performed on either $\left(X_{c},P_{c}\right)$ or $\left(X_{\mathrm{total}},P_{\mathrm{total}}\right)$, since they differ only by the known factor $\sqrt{N}$ in Equation (11).

### 3.5. Time-Resolved Homodyne Detection and Dynamical Fitting

Homodyne detection provides direct access to quadrature observables in CV systems and is routinely used in quantum optics to characterize Gaussian states and their dynamics [5,17]. By performing time-resolved homodyne measurements of the summed collective quadratures ${\hat{X}}_{\mathrm{total}}\left(t\right)$ and ${\hat{P}}_{\mathrm{total}}\left(t\right)$, and equivalently expressing them in canonically normalized form as ${\hat{X}}_{c}\left(t\right)$ and ${\hat{P}}_{c}\left(t\right)$ via $X_{c}\left(t\right)=X_{\mathrm{total}}\left(t\right)/\sqrt{N}$ and $P_{c}\left(t\right)=P_{\mathrm{total}}\left(t\right)/\sqrt{N}$ (Equation (11)), EMA enables estimation of the effective collective frequency $\Omega_{\mathrm{eff}}$ through dynamical fitting.

The quality of the effective description is assessed by comparing measured trajectories with the predictions of Equations (13) and (14), using statistical metrics such as residual analysis and goodness-of-fit measures. A close agreement supports the interpretation that the system’s collective behaviour is well captured by a single effective mode, even if the underlying microscopic dynamics remain complex. In practice, because $\left(X_{\mathrm{total}},P_{\mathrm{total}}\right)$ and $\left(X_{c},P_{c}\right)$ differ only by the known scale factor $\sqrt{N}$, fitting and residual analysis may be performed in either representation without changing inferred frequencies, while the EMA Hamiltonian is defined in the normalized coordinates.

In this way, EMA functions as a scalable, verification-oriented framework for assessing collective Hamiltonian behaviour in large multi-qu-mode systems. Its emphasis on experimentally accessible observables and statistical validation makes it particularly suitable for applied continuous-variable platforms, including photonic processors, cavity-based architectures, and engineered Gaussian networks.

## 4. Simulation Methods

This section describes the numerical simulation framework used to investigate collective dynamics in multi-qu-mode systems and to validate the Effective Mode Approximation (EMA) through time-resolved quadrature measurements. Rather than reconstructing microscopic Hamiltonian parameters mode by mode, the simulations are designed to infer and validate an effective collective Hamiltonian governing aggregate quadrature dynamics.

All simulations were performed using Xanadu’s Strawberry Fields platform with the Gaussian backend, which enables efficient and exact simulation of Gaussian continuous-variable (CV) quantum states and linear optical transformations [9]. This idealized setting excludes decoherence and technical noise, allowing direct assessment of EMA validity under controlled conditions.

### 4.1. System Size and Topology

We simulated systems comprising N=8,16,32, and 64 qu-modes. To ensure global mode connectivity and collective mixing, the interaction network was implemented using a ring topology, where each qu-mode interacts with its nearest neighbours via balanced beam-splitter operations. Local phase rotations were applied to each mode at every interaction layer to avoid trivial decoupling and to promote sustained collective dynamics.

The discrete simulation time index corresponds to the number of repeated interaction layers applied to the system, producing an effective time axis used for dynamical fitting.

### 4.2. Initial State Preparation

To enable meaningful inference of collective dynamics from first moments, the system is initialized using a probe-based strategy rather than vacuum-only states. Specifically, one or more selected qu-modes are prepared in a weak coherent state,

(15) $$|\alpha \rangle =\hat{D}\left(\alpha \right)|0\rangle ,$$

with controllable displacement amplitude and phase. In some configurations, optional single-mode squeezing is applied prior to displacement to enhance signal contrast.

This choice is deliberate. Under passive Gaussian evolution, an all-vacuum initial state preserves vanishing first moments, so a first-moment fit to $X_{c}\left(t\right)$ or $P_{c}\left(t\right)$ would be uninformative. Introducing a weak coherent probe creates a controlled nonzero signal in the collective quadratures while keeping the dynamics within the Gaussian regime [1,9]. All remaining qu-modes are initialized in the vacuum state.

### 4.3. Dynamical Evolution

Following state preparation, the system undergoes repeated application of the interaction layer, implementing collective mode mixing and phase accumulation. After a specified number of layers, the resulting Gaussian state is characterized by its mean vector and covariance matrix.

Rather than performing projective measurements within the simulator, homodyne measurement statistics are generated by sampling from the final Gaussian state. For a homodyne angle ϕ, the measured quadrature for each qu-mode is

(16) $${\hat{x}}_{\phi }=\hat{x}\cos\phi +\hat{p}\sin\phi .$$

For each run, quadrature outcomes are sampled across all qu-modes and aggregated to obtain both the experimentally natural summed observables and their canonically normalized collective counterparts. For homodyne angles ϕ=0 and ϕ=π/2, we compute

(17) $$X_{\mathrm{total}}\left(t\right)=\sum_{i=1}^{N}x_{i}\left(t\right),\;P_{\mathrm{total}}\left(t\right)=\sum_{i=1}^{N}p_{i}\left(t\right),$$

and define the normalized collective quadratures

(18) $$X_{c}\left(t\right)=\frac{1}{\sqrt{N}}X_{\mathrm{total}}\left(t\right),\;P_{c}\left(t\right)=\frac{1}{\sqrt{N}}P_{\mathrm{total}}\left(t\right).$$

This normalization ensures that the operators $\left({\hat{X}}_{c},{\hat{P}}_{c}\right)$ form a canonically conjugate pair satisfying $[{\hat{X}}_{c},{\hat{P}}_{c}]=i\hbar ,$ thereby enabling a consistent single-effective-oscillator description within the EMA framework. The summed observables $X_{\mathrm{total}},P_{\mathrm{total}}$ remain the directly interpretable experimental quantities, while $X_{c},P_{c}$ provide the properly normalized coordinates used for effective Hamiltonian inference.

In the present study, inference is performed from collective quadrature trajectories rather than full mode-resolved first- and second-moment data, which is why EMA is formulated as a reduced-order validation procedure rather than a full Gaussian-state reconstruction method.

### 4.4. Statistical Sampling and Averaging

To account for finite-sampling effects and to emulate realistic experimental acquisition, each configuration is repeated over multiple independent runs with distinct random seeds. For each run, a fixed number of homodyne samples (“shots”) is drawn from the underlying Gaussian state at each discrete time step. These samples generate empirical quadrature outcomes for all qu-modes, from which collective observables are constructed.

For each run and time index *t*, we compute the summed collective quadratures

(19) $$X_{\mathrm{total}}\left(t\right)=\sum_{i=1}^{N}x_{i}\left(t\right),\;P_{\mathrm{total}}\left(t\right)=\sum_{i=1}^{N}p_{i}\left(t\right),$$

and, when required for canonical consistency, their normalized counterparts

(20) $$X_{c}\left(t\right)=\frac{1}{\sqrt{N}}X_{\mathrm{total}}\left(t\right),\;P_{c}\left(t\right)=\frac{1}{\sqrt{N}}P_{\mathrm{total}}\left(t\right).$$

Within each run, shot-level samples are averaged to produce run-level estimates of the collective quadrature means. These run-level means are then averaged across independent runs to obtain ensemble-level trajectories $\langle X_{\mathrm{total}}\left(t\right)\rangle$ and $\langle P_{\mathrm{total}}\left(t\right)\rangle .$ The corresponding empirical standard deviations across runs quantify finite-sampling variability and provide uncertainty bands for visualization and fitting.

This hierarchical averaging procedure separates shot noise (within-run fluctuations) from run-to-run statistical variability, yielding robust estimates of collective dynamics suitable for effective-mode inference. The resulting ensemble-level trajectories form the basis for sinusoidal fitting, residual analysis, and model-selection procedures within the EMA validation pipeline.

### 4.5. Effective-Mode Fitting and Validation

The ensemble-averaged collective quadratures are fitted to sinusoidal models derived from the EMA effective Hamiltonian written in terms of the canonically normalized collective mode,

(21) $${\hat{H}}_{\mathrm{eff}}=\frac{\hbar \Omega_{\mathrm{eff}}}{2}\left({\hat{X}}_{c}^{2}+{\hat{P}}_{c}^{2}\right),$$

yielding estimates of the effective oscillation frequency $\Omega_{\mathrm{eff}}$ along with nuisance parameters (amplitude, phase, and offset). The fitted dynamical models are evaluated against time-resolved trajectories obtained from the simulation ensemble.

In practice, the fitting and residual analysis may be performed equivalently on the normalized trajectories $\left\{X_{c}\left(t\right),P_{c}\left(t\right)\right\}$ or on the summed trajectories $\left\{X_{\mathrm{total}}\left(t\right),P_{\mathrm{total}}\left(t\right)\right\}$, since they differ only by the known scale factor $\sqrt{N}$ (Equation (11)). We report results using the experimentally natural summed observables for interpretability, while the EMA model definition and parameter inference are grounded in the normalized collective mode.

Model quality is assessed using residual analysis and goodness-of-fit metrics, enabling quantitative evaluation of EMA validity across system sizes. By design, this methodology tests whether a single effective collective mode can accurately reproduce the observed aggregate quadrature dynamics, even as the number of physical qu-modes increases. In the present study, EMA is regarded as supported when three conditions are met simultaneously: the fitted model reproduces the dominant oscillatory trajectory, residuals remain statistically centred near zero without systematic drift, and a more complex harmonic model is not preferred by AIC except where explicitly reported. This criterion emphasizes validation of dominant collective behaviour rather than exhaustive recovery of all microscopic degrees of freedom. The resulting agreement between simulation data and EMA predictions provides a direct validation of EMA as a scalable modelling and inference framework for large continuous-variable quantum systems.

## 5. Results

This section presents numerical results validating the Effective Mode Approximation (EMA) using time-resolved collective quadrature measurements obtained from Gaussian simulations. Rather than reconstructing microscopic Hamiltonian parameters, the analysis focuses on whether a single effective collective mode can accurately reproduce the observed ensemble-averaged dynamics across increasing system sizes.

All results are based on probe-initialized multi-qu-mode systems simulated using the Gaussian backend of Xanadu’s Strawberry Fields platform. Collective quadratures $X_{\mathrm{total}}$ and $P_{\mathrm{total}}$ are extracted from homodyne-sampled Gaussian states and compared against EMA-derived dynamical models.

### 5.1. Simulation Results and Data Analysis

Simulations were performed for systems comprising N=8,16,32, and 64 qu-modes. For each configuration, collective quadratures $X_{\mathrm{total}}$ and $P_{\mathrm{total}}$ were evaluated over multiple independent runs and averaged to obtain ensemble-level trajectories. The corresponding standard deviations quantify finite-sampling fluctuations and provide uncertainty estimates for model fitting.

The time evolution of the collective quadrature $X_{\mathrm{total}}$ exhibits clear oscillatory behaviour consistent with effective harmonic dynamics, with increasing signal strength and improved stability as the number of qu-modes grows.

The plots below are shown for one representative simulation batch generated on 22 January 2026 at 08:27:50 (UTC), using a fixed configuration of system parameters and random seeds. This choice keeps the visual presentation compact; the same fitting and model-selection pipeline was applied consistently across all simulated system sizes, and the quantitative metrics reported in Table 1 summarize the corresponding best-fit behaviour.

#### 5.1.1. Collective Quadrature Evolution

**Figure 1:**
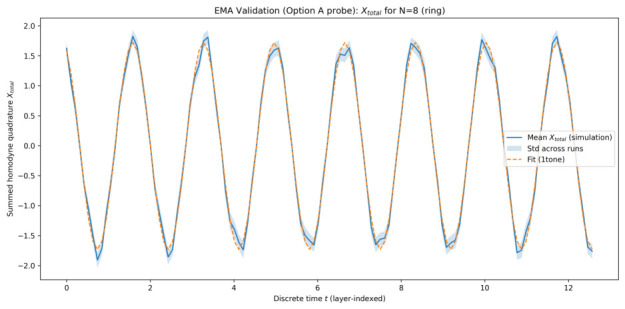
Time evolution of $X_{\mathrm{total}}$ for 8 qu-modes as a function of discrete time *t* (layer index). Solid curve: ensemble-averaged simulation results. Shaded region: one standard deviation across runs. Dashed curve: best-fit EMA model for the representative batch.

**Figure 2:**
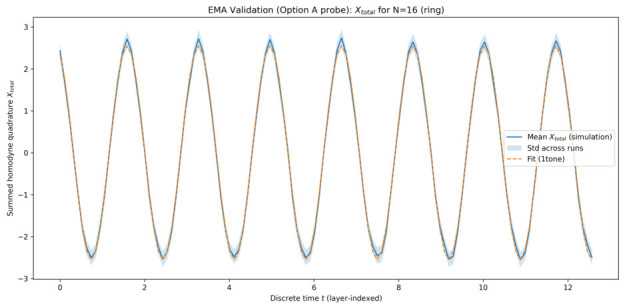
Time evolution of $X_{\mathrm{total}}$ for 16 qu-modes as a function of discrete time *t* (layer index). Solid curve: ensemble-averaged simulation results. Shaded region: one standard deviation across runs. Dashed curve: best-fit EMA model for the representative batch.

**Figure 3:**
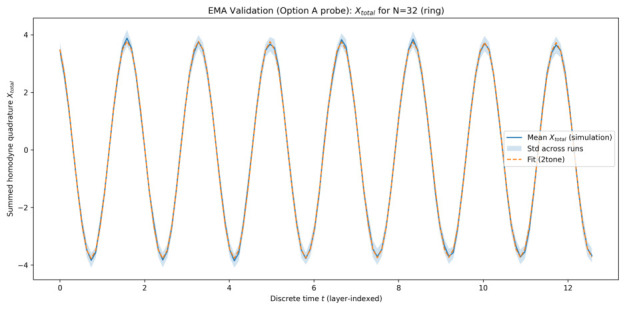
Time evolution of $X_{\mathrm{total}}$ for 32 qu-modes as a function of discrete time *t* (layer index). Solid curve: ensemble-averaged simulation results. Shaded region: one standard deviation across runs. Dashed curve: best-fit EMA model for the representative batch.

**Figure 4:**
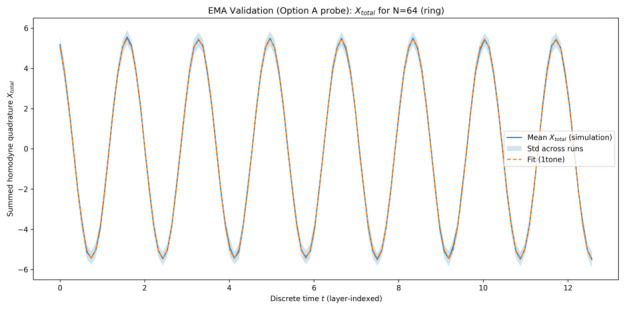
Time evolution of $X_{\mathrm{total}}$ for 64 qu-modes as a function of discrete time *t* (layer index). Solid curve: ensemble-averaged simulation results. Shaded region: one standard deviation across runs. Dashed curve: best-fit EMA model for the representative batch.

Figure 1, Figure 2, Figure 3 and Figure 4 show the time evolution of the ensemble-averaged collective quadrature $X_{\mathrm{total}}$ for systems with 8, 16, 32, and 64 qu-modes, respectively. In each figure, the solid curve represents the mean trajectory across runs, while the shaded region indicates one standard deviation. The dashed curve corresponds to the best-fit EMA model obtained from sinusoidal fitting. The horizontal axis is the discrete layer index used as the effective time coordinate, and the vertical axis is the summed collective quadrature. Presenting the raw summed observable $X_{\mathrm{total}}$ preserves direct experimental interpretability, while the fitted EMA parameters remain equivalent to those obtained in normalized coordinates up to the known $\sqrt{N}$ scaling.

Across all system sizes, the EMA model accurately captures the dominant oscillatory dynamics of the collective quadrature. Minor deviations are observable at early times and for smaller systems, where finite-size effects and mode-specific contributions are more pronounced. As the number of qu-modes increases, the agreement between simulation data and the effective-mode model improves, indicating enhanced collective behaviour.

The narrowing of relative fluctuations and improved fit quality at larger *N* indicates that collective averaging suppresses mode-specific noise, making the effective single-mode description increasingly accurate.

#### 5.1.2. Residual Analysis

**Figure 5:**
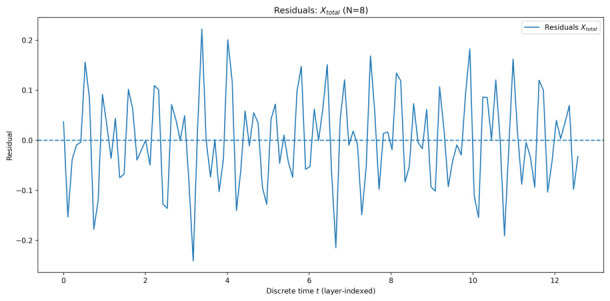
Residuals of $X_{\mathrm{total}}$ for 8 qu-modes as a function of discrete time *t* (layer index), defined as simulation minus the AIC-selected EMA fit.

**Figure 6:**
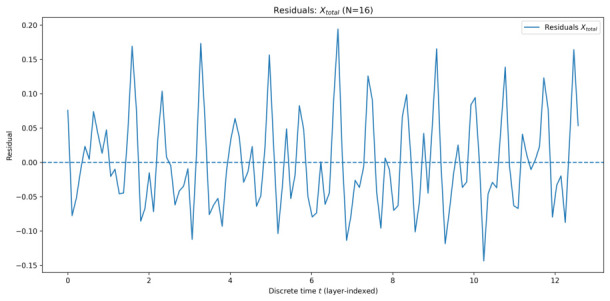
Residuals of $X_{\mathrm{total}}$ for 16 qu-modes as a function of discrete time *t* (layer index), defined as simulation minus the AIC-selected EMA fit.

**Figure 7:**
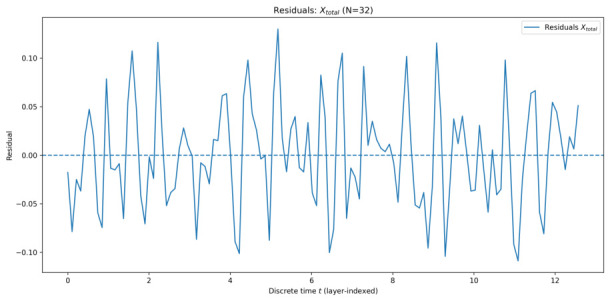
Residuals of $X_{\mathrm{total}}$ for 32 qu-modes as a function of discrete time *t* (layer index), defined as simulation minus the AIC-selected EMA fit.

**Figure 8:**
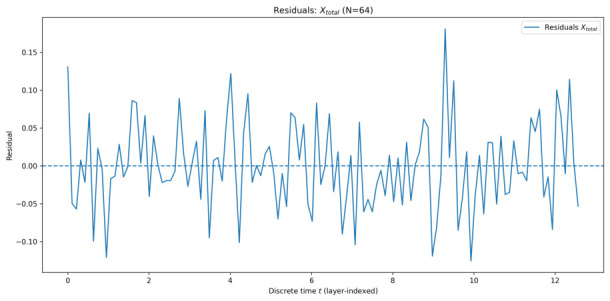
Residuals of $X_{\mathrm{total}}$ for 64 qu-modes as a function of discrete time *t* (layer index), defined as simulation minus the AIC-selected EMA fit.

To quantitatively assess the quality of the EMA description, residuals were computed as the difference between the ensemble-averaged simulation data and the corresponding EMA-based fit. Figure 5, Figure 6, Figure 7 and Figure 8 show the residuals for $X_{\mathrm{total}}$ across all system sizes.

In all cases, residuals remain centred around zero with no systematic drift, confirming that the dominant dynamics are well captured by the effective-mode model. For smaller systems, residuals exhibit weak structured fluctuations, reflecting incomplete collective mixing and residual multi-mode effects. As the number of qu-modes increases, residual amplitudes decrease and approach noise-like behaviour, consistent with convergence toward a collective effective mode.

Overall, the residual analysis supports the central claim of EMA: as system size increases, collective quadrature dynamics become increasingly well-described by a single effective mode, despite the underlying high-dimensional structure of the system.

### 5.2. Fitted Parameters for Collective Quadrature Dynamics

To quantitatively assess the validity of the Effective Mode Approximation (EMA), the ensemble-averaged collective quadratures were fitted to effective harmonic models. Rather than assuming a fixed single-frequency description a priori, both one-tone and two-tone sinusoidal models were considered. Model selection was performed using the Akaike Information Criterion (AIC), allowing the data to determine whether residual multi-mode contributions were statistically significant.

For each system size, the collective quadratures $X_{\mathrm{total}}\left(t\right)$ and $P_{\mathrm{total}}\left(t\right)$ were fitted independently. Initial frequency estimates were obtained via fast Fourier transform (FFT) analysis, followed by non-linear least-squares optimization. Fit quality was quantified using the root-mean-square error (RMSE), normalized RMSE (NRMSE), and coefficient of determination $R^{2}$.

Table 1 summarizes the dominant effective frequencies extracted from the best-fitting models, along with associated goodness-of-fit metrics for the collective $X_{\mathrm{total}}$ quadrature.

Across all system sizes, the dominant effective frequency remains remarkably stable, indicating that the collective dynamics are governed by a well-defined emergent mode. For the intermediate case N=32, AIC weakly prefers a two-tone model. We interpret this not as a failure of EMA, but as evidence that finite-size or symmetry-breaking effects can place the data near the boundary between a strictly single-mode response and a weak residual multi-mode correction. By N=64, the single-tone description again dominates.

The monotonic improvement in RMSE, NRMSE, and $R^{2}$ with increasing system size demonstrates convergence toward an effective single-mode description. This behaviour supports the central premise of EMA: as the number of interacting qu-modes increases, microscopic details become increasingly irrelevant, and collective observables are accurately captured by a low-dimensional effective Hamiltonian.

### 5.3. Evaluation of the Effective Mode Approximation (EMA)

The results obtained from Gaussian simulations provide strong quantitative evidence supporting the validity of the Effective Mode Approximation (EMA) across a broad range of system sizes. For all configurations considered (N=8,16,32,64), the ensemble-averaged collective quadratures $X_{\mathrm{total}}\left(t\right)$ and $P_{\mathrm{total}}\left(t\right)$ closely follow the dynamics predicted by an effective low-dimensional Hamiltonian, as evidenced by consistently high coefficients of determination ($R^{2}>0.99$) and low normalized root-mean-square errors.

A key observation is the stability of the dominant effective frequency across increasing numbers of qu-modes. Despite the exponential growth of the underlying Hilbert space and the presence of many interacting modes, the extracted effective frequency remains stable within statistical tolerance across system sizes, indicating that the collective dynamics are governed by a well-defined emergent mode. This behaviour directly supports the central premise of EMA: microscopic mode-specific details become progressively less relevant as collective observables dominate the system’s evolution.

Residual analysis further reinforces this conclusion. Across all system sizes, residuals between simulated trajectories and fitted effective models remain centred around zero and exhibit no systematic drift, indicating that deviations are statistical rather than structural. While intermediate system sizes (notably N=32) exhibit weak multi-tone features, model selection via the Akaike Information Criterion confirms that these contributions are subdominant and become increasingly suppressed at larger *N*. In the largest system studied (N=64), the data strongly favour a single-tone description, demonstrating convergence toward an effectively single-mode regime.

From a computational perspective, EMA achieves a substantial reduction in modelling complexity. Rather than requiring full mode-by-mode Hamiltonian reconstruction, the collective behaviour of systems with up to 64 interacting qu-modes is accurately captured using only a small number of effective parameters. This reduction enables scalable validation of large continuous-variable quantum systems using experimentally accessible observables, without sacrificing predictive accuracy.

These results should be interpreted within the scope of the present validation task. They show that EMA captures the dominant collective behaviour accessible through summed quadratures under ideal Gaussian dynamics; they do not imply that EMA reconstructs the full multi-mode state or supersedes more expressive state-representation methods.

Taken together, these results support EMA as a robust and scalable approximation framework for inferring and validating collective Hamiltonians in large, entangled or symmetrically coupled multi-qu-mode systems. The observed convergence toward effective single-mode dynamics highlights EMA’s suitability for high-dimensional continuous-variable platforms where traditional reconstruction techniques become computationally impractical.

## 6. Discussion and Conclusions

### 6.1. Interpretation of Key Results

The results presented in this study demonstrate that the Effective Mode Approximation (EMA) provides a robust and scalable framework for modelling the collective dynamics of multi-qu-mode systems, particularly in regimes characterized by strong entanglement or high symmetry. Across all investigated system sizes (N=8,16,32, and 64), the collective quadrature dynamics predicted by the EMA-based effective Hamiltonian exhibit close agreement with Gaussian simulation data, as evidenced by consistently small residuals and high goodness-of-fit metrics.

A central observation is that the dominant collective behaviour of the system can be accurately captured using a reduced, low-dimensional description, despite the exponential growth of the underlying Hilbert space with increasing *N*. The residuals between simulated and fitted trajectories remain centred around zero with no systematic temporal structure, indicating that discrepancies arise primarily from statistical fluctuations rather than from unmodelled dynamical effects. This behaviour confirms that the EMA successfully isolates the leading collective mode governing the system’s evolution.

The dominant effective frequency remains stable across system sizes, while model selection indicates that residual corrections are weak and largely confined to the intermediate N=32 case. This behaviour suggests that the emergent collective description scales smoothly with system size rather than exhibiting instability or divergence. In that sense, EMA captures a system-level description of the dominant observed dynamics rather than artifacts tied to a single microscopic realization.

Taken together, these findings indicate that the Effective Mode Approximation (EMA) is not merely a numerical fitting convenience but a physically meaningful reduced-order inference framework that reflects genuine collective behaviour in large continuous-variable quantum systems. Rather than attempting exhaustive microscopic reconstruction, EMA tests whether experimentally accessible collective observables are statistically consistent with an effective single-mode Hamiltonian description. By suppressing the need for detailed mode-by-mode modelling while preserving dynamical accuracy, EMA provides a scalable and computationally efficient pathway for inferring and validating collective Hamiltonian behaviour in high-dimensional quantum platforms where traditional reconstruction methods become computationally infeasible.

### 6.2. Comparison with Existing Research

The Effective Mode Approximation (EMA) builds upon a substantial body of work in continuous-variable (CV) quantum mechanics, particularly research on Gaussian quantum states, collective observables, and effective Hamiltonian descriptions [1,2]. Traditional Hamiltonian reconstruction techniques in multi-qu-mode systems typically rely on detailed, mode-resolved modelling of local dynamics and pairwise interactions. While such approaches are theoretically well-founded, their computational cost grows rapidly with system size, rendering them impractical for large, highly entangled, or densely connected systems.

In contrast, the EMA framework introduced here departs from mode-resolved reconstruction by explicitly targeting the dominant collective dynamics accessible through experimentally relevant observables, namely collective quadrature measurements. Rather than retaining a reduced but still multi-mode Hamiltonian, EMA collapses the system description to a single effective mode whose dynamics encode the aggregate behaviour of the full system. This reduction is not imposed at the level of microscopic assumptions, but instead emerges from the observed collective dynamics, distinguishing EMA from conventional truncation or perturbative approaches.

Conceptually, EMA is related to effective Hamiltonian and mode-selective interaction techniques developed in quantum optics and open quantum systems [4,7,16]. These methods aim to simplify complex dynamics by isolating relevant subspaces or interaction channels. However, existing approaches typically preserve an explicit multi-mode structure, even in reduced form, and are often derived under restrictive assumptions about coupling hierarchies or weak interactions. EMA advances beyond these methods by demonstrating that, in regimes of strong symmetry or entanglement, the system’s observable dynamics can be faithfully represented by a single collective degree of freedom without explicit reference to individual modes.

Related collective models, such as the Dicke model and its generalizations [18], also emphasize collective behaviour, but remain fundamentally multi-component descriptions in which microscopic constituents retain explicit representation. By contrast, EMA provides a phenomenological yet quantitatively accurate single-mode description derived directly from collective measurement statistics. This distinction is particularly relevant from an engineering and verification perspective, where access to individual mode information may be limited or unnecessary.

Effective descriptions have also proven valuable in fields such as Bose–Einstein condensates and cavity quantum electrodynamics, where collective variables capture macroscopic behaviour [8,19]. EMA complements these approaches by offering a measurement-driven, reconstruction-oriented framework tailored to continuous-variable quantum systems and scalable architectures. Importantly, EMA is validated across increasing system sizes without a corresponding growth in model complexity, highlighting its suitability for large-scale implementations.

Overall, EMA should be viewed as a focused methodological contribution to Hamiltonian verification in CV quantum systems. By prioritizing collective observables and emergent dynamics over microscopic detail, EMA offers a computationally efficient and experimentally aligned pathway for characterizing large, entangled systems when full reconstruction is unnecessary or impractical.

### 6.3. Comparison with Tensor-Network Approaches

A natural comparison point for EMA is the family of tensor-network state methods. Projected entangled pair states (PEPS) extend matrix–product–state constructions beyond one dimension, and continuous projected entangled pair states (cPEPS) extend the same logic to quantum fields [14,15]. These methods are substantially more expressive than EMA because they aim to represent the full state, or a controlled approximation to it, rather than only a reduced set of collective observables.

The relevant notion of efficiency is therefore task dependent. PEPS-type methods trade additional computational cost, through variational optimization, tensor contraction, and bond-dimension control, for access to mode-resolved or spatially structured information. EMA, by contrast, fits a fixed number of effective parameters directly to homodyne-accessible summed quadratures and entirely avoids full-state optimization. For the narrower task considered here, namely validating dominant collective Hamiltonian behaviour from aggregate measurements, EMA is therefore computationally lighter, but it is also correspondingly less expressive.

These approaches should thus be viewed as complementary rather than competing. Tensor-network methods are preferable when the scientific goal is state representation, simulation, or entanglement-resolved analysis. EMA is preferable when the goal is rapid verification that experimentally accessible collective dynamics are consistent with a low-dimensional effective Hamiltonian. An important future benchmark will be to compare EMA against tensor-network surrogates in partially symmetry-broken regimes where collective and mode-resolved structure coexist.

### 6.4. Broader Implications for Quantum Information Processing

The validation of the Effective Mode Approximation (EMA) as a robust framework for reduced-order effective Hamiltonian inference carries important implications for quantum information processing, particularly in scalable continuous-variable (CV) architectures. By enabling accurate characterization of system-wide dynamics without reliance on detailed, mode-resolved descriptions, EMA provides a practical pathway for monitoring and verifying large, highly entangled quantum systems where conventional reconstruction techniques become infeasible.

In CV quantum computing and communication platforms, where information is often encoded and processed collectively across many modes, EMA offers a natural tool for assessing global system behaviour using experimentally accessible observables. Quantum protocols such as entanglement-based quantum cryptography, quantum teleportation, and CV error correction schemes could benefit from EMA by allowing real-time verification of collective dynamics and effective frequencies, reducing the overhead associated with tracking individual mode contributions. This feature is particularly relevant for large-scale or dynamically reconfigurable systems, where full mode tomography is impractical.

Beyond computation and communication, EMA also has clear relevance for quantum simulation. Many-body quantum simulators implemented in optical cavities, photonic lattices, or multi-mode optical platforms aim to reproduce emergent collective phenomena rather than microscopic details. In this context, EMA provides a computationally efficient means of modelling and validating collective excitations, effective couplings, and global dynamical properties. By focusing on emergent behaviour captured through collective quadratures, EMA supports the development of scalable, resource-efficient quantum simulators while maintaining quantitative agreement with underlying dynamics.

More broadly, EMA aligns with a growing emphasis on verification-oriented and device-agnostic approaches in quantum technologies, where the goal is to certify system behaviour through limited, high-level measurements rather than exhaustive internal access. As CV quantum systems continue to scale in size and complexity, frameworks such as EMA will be increasingly valuable for bridging the gap between theoretical models, numerical simulations, and experimentally observable dynamics in practical quantum information processing platforms.

### 6.5. Limitations

While the Effective Mode Approximation (EMA) demonstrates strong potential for modelling collective dynamics in multi-qu-mode systems, it is subject to well-defined limitations that delimit its domain of applicability. Most notably, EMA is most effective in regimes where dominant collective observables capture the leading system dynamics, such as in symmetric or strongly correlated configurations, collective coupling, or global entanglement such that a small number of emergent degrees of freedom dominate the observed dynamics. In systems where interactions between qu-modes are highly irregular, strongly localized, or explicitly engineered to break symmetry, the collective description provided by EMA may no longer capture the full dynamical behaviour.

Additionally, EMA is designed to model dominant, system-wide dynamics rather than fine-grained, mode-resolved effects. As a consequence, weak local interactions, mode-dependent disorder, or subtle correlations that do not significantly contribute to collective observables may be effectively averaged out. While this is an intentional feature of the approximation, aligned with its goal of scalability and computational efficiency, it may limit accuracy in scenarios where detailed mode-specific information is essential, such as precision control of individual qu-modes or highly heterogeneous coupling networks.

For the same reason, EMA is not intended to replace full-state ansatz such as PEPS or cPEPS when the target output is state reconstruction or entanglement-resolved analysis [14,15].

A further limitation of the present framework is its reliance on Gaussian states and linear optical interactions. The simulations and analytical treatment presented here assume Gaussian dynamics, which are well-suited to many continuous-variable (CV) platforms but do not encompass the full range of physically relevant quantum states. Non-Gaussian states, strong non-linearities, and measurement-induced non-Gaussian effects play an increasingly important role in advanced quantum information processing and may not be faithfully captured by the current EMA formulation. Extending EMA to incorporate non-Gaussian features or effective descriptions of non-linear dynamics represents a natural and important direction for future work.

Accordingly, the present validation should be interpreted as confined to the Gaussian/linear regime considered here. Extending EMA to non-Gaussian or strongly non-linear settings would require higher-order observable summaries and corresponding inference models.

Finally, EMA should be understood as a verification- and modelling-oriented framework rather than a complete substitute for full Hamiltonian reconstruction. Its strength lies in providing reliable, system-level insight when exhaustive reconstruction is impractical, rather than in resolving microscopic details. Within this scope, EMA offers a controlled and transparent approximation whose limitations are well understood and can be explicitly tested against more detailed models when required.

### 6.6. Future Directions

Several promising research directions emerge naturally from the Effective Mode Approximation (EMA) framework presented in this work. A primary avenue for future investigation is the extension of EMA beyond Gaussian states and linear interactions. While Gaussian dynamics capture a wide class of experimentally relevant continuous-variable (CV) systems, non-Gaussian states and non-linear processes are increasingly central to quantum computation, quantum metrology, and fault-tolerant architectures. Developing generalized EMA formulations that incorporate effective non-linearities or reduced non-Gaussian descriptors would significantly broaden the framework’s applicability and deepen its relevance to advanced quantum technologies.

A second important direction is the experimental validation of EMA in realistic laboratory settings. The results presented here rely on idealized Gaussian simulations that neglect noise, loss, and decoherence. Implementing EMA-based reconstruction protocols in physical platforms, such as optical cavities, integrated photonic circuits, Bose–Einstein condensates, or microwave CV systems, would provide critical insight into the framework’s robustness under experimental imperfections. Such studies could also clarify the relationship between collective observables and effective Hamiltonian parameters in the presence of dissipation, mode mismatch, and finite measurement resolution.

Another promising avenue involves integrating EMA with data-driven and machine learning-based parameter estimation techniques. Given that EMA reduces high-dimensional system dynamics to a small number of effective parameters, it is particularly well-suited for hybrid approaches where classical learning algorithms assist in extracting collective frequencies, coupling strengths, or stability indicators from time-resolved measurement data. Machine learning methods trained on simulated or experimental datasets could accelerate parameter inference, improve robustness to noise, and enable real-time monitoring of collective dynamics in large-scale CV systems.

A complementary direction is to benchmark EMA against tensor-network surrogates, using PEPS/cPEPS-style state representations as richer references in regimes with partial symmetry breaking or stronger a multi-mode structure [14,15]. Such comparisons would help quantify more precisely when the additional expressive power of a full-state ansatz becomes necessary and when the lighter-weight EMA description remains sufficient.

Finally, future work may explore the role of EMA as a diagnostic or verification tool rather than a full reconstruction method. In this context, EMA could serve as a lightweight consistency check for complex quantum devices, providing rapid validation of collective behaviour without requiring exhaustive tomography. Such applications are particularly relevant for scalable and device-independent quantum architectures, where verifying global system properties is often more practical, and more meaningful, than reconstructing detailed microscopic models.

Together, these directions position EMA as a flexible and extensible framework with potential impact across quantum optics, continuous-variable quantum information processing, and the characterization of large-scale entangled quantum systems.

## Figures and Tables

**Table 1:** Best-fit effective frequencies and goodness-of-fit metrics for the collective quadrature $X_{\mathrm{total}}$. Model selection was performed using the Akaike Information Criterion (AIC); for N=32, both frequencies of the preferred two-tone fit are reported. Frequencies are reported in dimensionless simulation units.

Qu-Modes	Model	$\omega_{\mathrm{eff}}$	RMSE	NRMSE	$R^{2}$
8	1-tone	3.97	$9.16\times 10^{-2}$	$2.46\times 10^{-2}$	0.994
16	1-tone	3.97	$7.03\times 10^{-2}$	$1.33\times 10^{-2}$	0.999
32	2-tone	3.97, 3.47	$5.41\times 10^{-2}$	$6.99\times 10^{-3}$	0.9996
64	1-tone	3.97	$5.75\times 10^{-2}$	$5.20\times 10^{-3}$	0.9998

## Data Availability

Data available from the corresponding author upon reasonable request.

## Abbreviations

The following abbreviations are used in this manuscript:

AIC	Akaike Information Criterion
BEC	Bose–Einstein Condensate
CV	Continuous Variable
DV	Discrete Variable
EMA	Effective Mode Approximation
EPR	Einstein–Podolsky–Rosen
FFT	Fast Fourier Transform
NRMSE	Normalized Root Mean Square Error
QIP	Quantum Information Processing
RMSE	Root Mean Square Error
PEPS	Projected Entangled Pair States
cPEPS	Continuous Projected Entangled Pair States
